# Supplementary material for: Massive Multiplexing of Spatially Resolved Single Neuron Projections with Axonal BARseq
Source: bioRxiv. 2023 Feb 18:2023.02.18.528865. Preprint. [Version 1] doi: 10.1101/2023.02.18.528865 (PMC9949159; doi:10.1101/2023.02.18.528865)
Supplement: 4 [file NIHPP2023.02.18.528865v1-supplement-4.pdf]

bioRxiv preprint doi: https://doi.org/10.1101/2023.02.18.528865; this version posted February 18, 2023. The copyright holder for this preprint (which was not certified by peer review) is the author/funder, who has granted bioRxiv a license to display the preprint in perpetuity. It is made available under aCC-BY 4.0 International license.

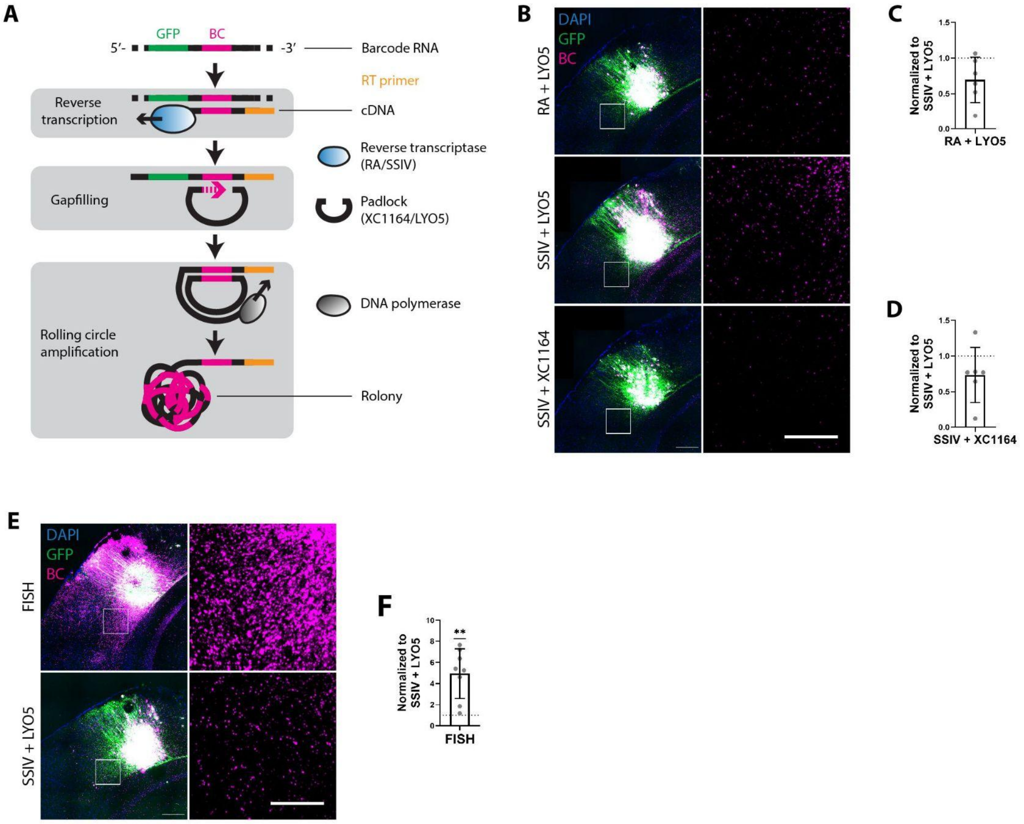

SupFig. 1 Optimization and efficiency estimation of axonal rolony preparation. (**A**) A brief summary of rolony preparation. Barcoded RNAs in axons and somata are first reverse transcribed into cDNA using reverse transcriptase. A padlock probe is then used to bind to the cDNA through adjacent regions of the barcode. The gap in the padlock is filled in using DNA polymerase, forming a circular single-stranded DNA molecule (ssDNA) through ligation by ligase. The circular ssDNA serves as a template for rolling circle amplification, resulting in the production of multiple copies of the barcode in a cluster of ssDNA called rolony. (**B-D**) Improvement of axonal rolony density using modified rolony preparation protocol. RA (RevertAid H Minus reverse transcriptase) and padlock probe XC1164 were used for reverse transcription and gap-filling for BaristaSeq (X. Chen et al. 2018). In the modified protocol, SSIV (SuperScript IV) and padlock probe LYO5 were used for reverse transcription and gap-filling. (**B**) Representative images of axonal rolonies using different rolony preparation conditions. Images were taken around the injection site in primary auditory cortex (AudI). Barcoded cells expressed both barcode RNA and GFP protein. (**C-D**) Both modifications yielded higher axonal rolonies density (one sample *t*-test). Each data point is the median of normalized rolony density from one brain section, two sections per mouse, total 3 mice. (**E-F**) The density of axonal barcodes was compared using two methods: FISH (RNAscope) and rolony preparation. Each barcoded RNA molecule has one GFP and one barcode. FISH detects the GFP component in the barcode RNA, while rolony preparation detects the barcode component. (**E**) Representative images of axonal barcode RNA around the injection site in AudI. (**F**) Comparison of the RNA density using FISH and the modified rolony preparation protocol (one sample *t*-test). Each data point is the median of normalized rolony density from one brain section, two sections per mouse, total 4 mice. Scale bar: top, 250 μm; bottom, 100 μm.

bioRxiv preprint doi: https://doi.org/10.1101/2023.02.18.528865; this version posted February 18, 2023. The copyright holder for this preprint (which was not certified by peer review) is the author/funder, who has granted bioRxiv a license to display the preprint in perpetuity. It is made available under aCC-BY 4.0 International license.

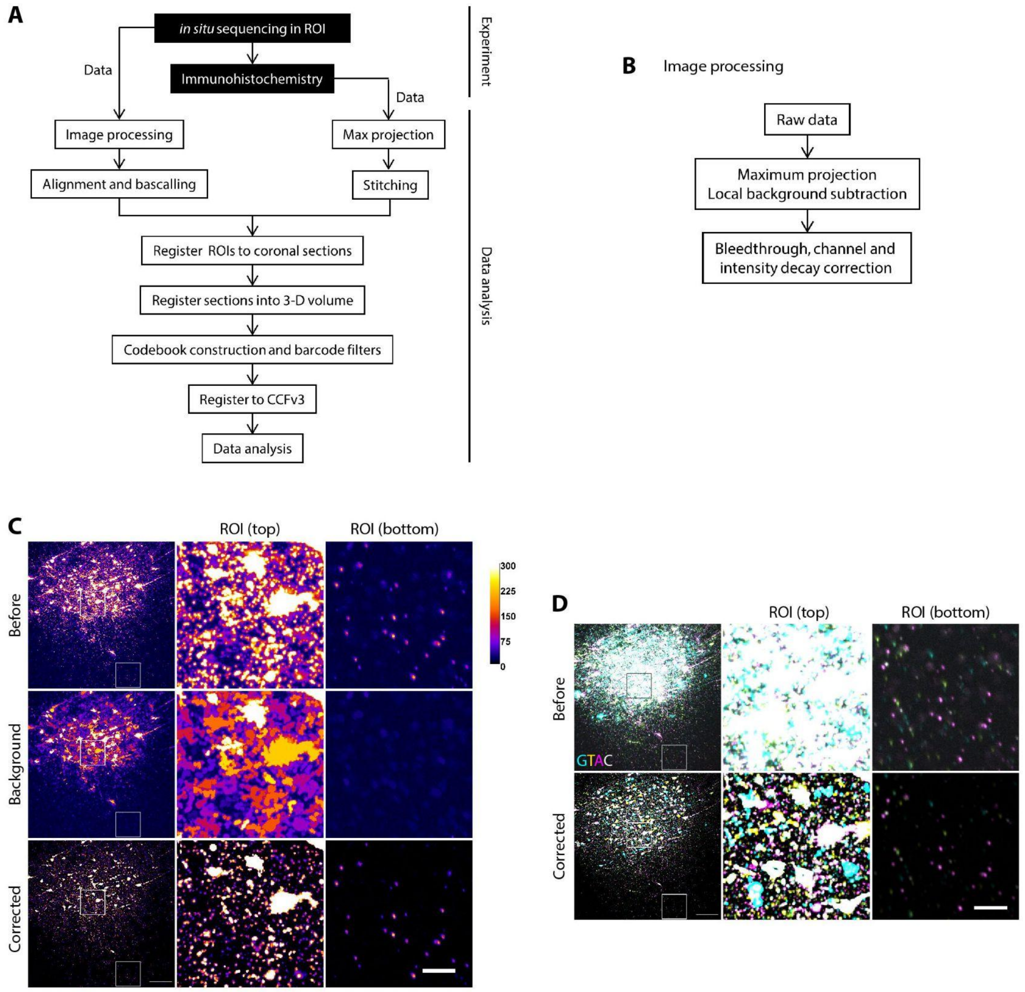

SupFig. 2 Data processing workflow and imaging processing. (**A**) The workflow of data acquisition and analysis for axonal BARseq. Additional information on image processing, alignment, basecalling, codebook construction, and barcode filtering can be found in SupFig 2-4. (**B**) The workflow of image processing. Raw data were acquired as z-stacks and then converted into maximum projections with background subtraction. Rolony intensity was then detected and used for further correction including bleedthrough, unequal channel intensity, and intensity decay during *in situ* sequencing. (**C-D**) Background subtraction effectively reduced the impact of uneven illumination, tissue and nucleus background on the images. The pixel intensity in the corrected images represented the signal beyond the background and was used for downstream analysis. Representative single tile from AudI, comparison of (**C**) single channel and (**D**) all four channels before and after background subtraction. Scale bar, 25 µm; color bar, single channel intensity.

bioRxiv preprint doi: https://doi.org/10.1101/2023.02.18.528865; this version posted February 18, 2023. The copyright holder for this preprint (which was not certified by peer review) is the author/funder, who has granted bioRxiv a license to display the preprint in perpetuity. It is made available under aCC-BY 4.0 International license.

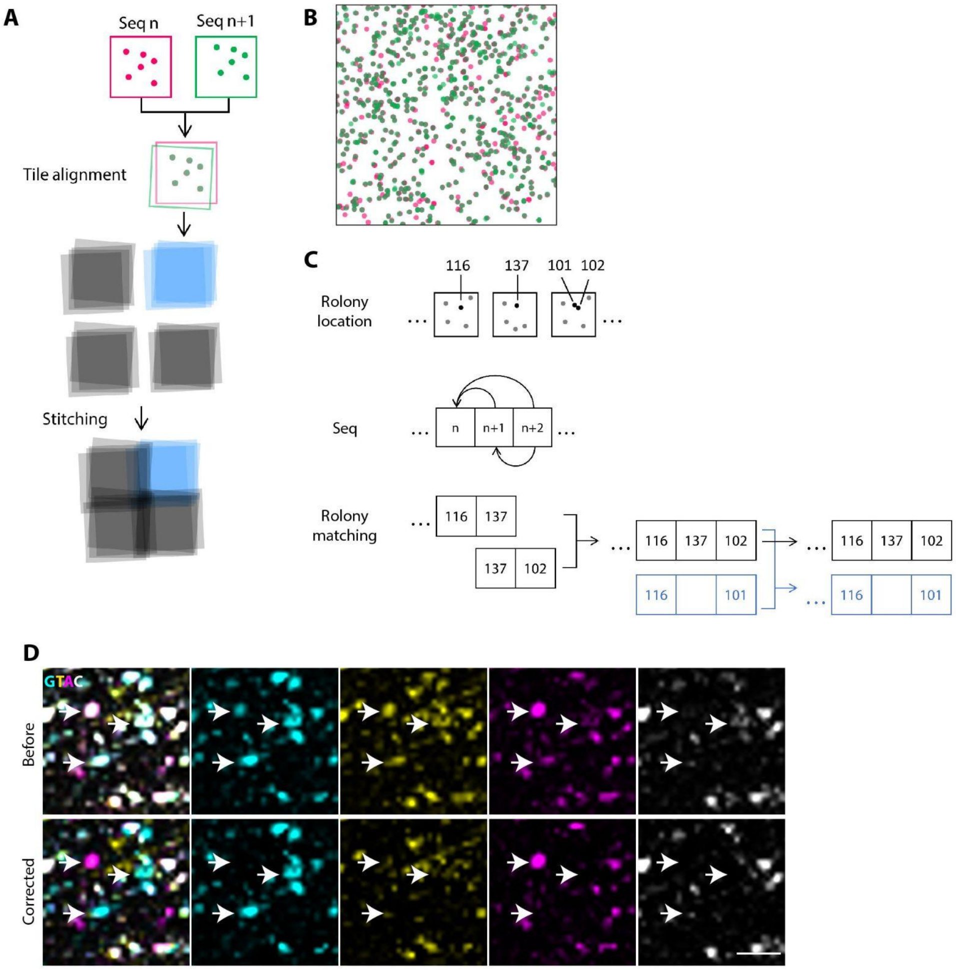

SupFig. 3 The processing steps for alignment, stitching and basecalling. (**A**) The workflow of alignment and stitching for axonal BARseq. Rolonies were identified as local maxima in individual tiles for each sequencing cycle. Tiles were first aligned across cycles using the locations of the rolonies, and then the aligned tile stacks were stitched together using the locations of the rolonies in the overlap regions. (**B**) Example of point cloud registration between neighboring sequencing cycles (magenta and green). 400 × 400-pixel area from AudI. (**C**) The workflow of rolony basecalling. During each sequencing cycle, unique IDs were assigned to individual rolonies (top row). Rolonies were then matched to rolonies in multiple previous sequencing cycles (middle row). For each match, a pair of IDs was identified, and pairs from different matches were merged or branched into an additional sequence (blue). Barcode sequences were assembled using the nucleotide associated with each ID. If no ID was found in a particular cycle, no nucleotide was assigned and the grid remains empty. (**D**) Representative images for non-specific signal correction in somata. Images were selected from Seq11 in AudI. Most of the somata had T in the previous sequencing cycle due to fixed barcode digits, and the signal was carried over to this cycle (top row). The correction effectively reduces the non-specific signals (bottom row). Scale bar, 50 µm.

bioRxiv preprint doi: https://doi.org/10.1101/2023.02.18.528865; this version posted February 18, 2023. The copyright holder for this preprint (which was not certified by peer review) is the author/funder, who has granted bioRxiv a license to display the preprint in perpetuity. It is made available under aCC-BY 4.0 International license.

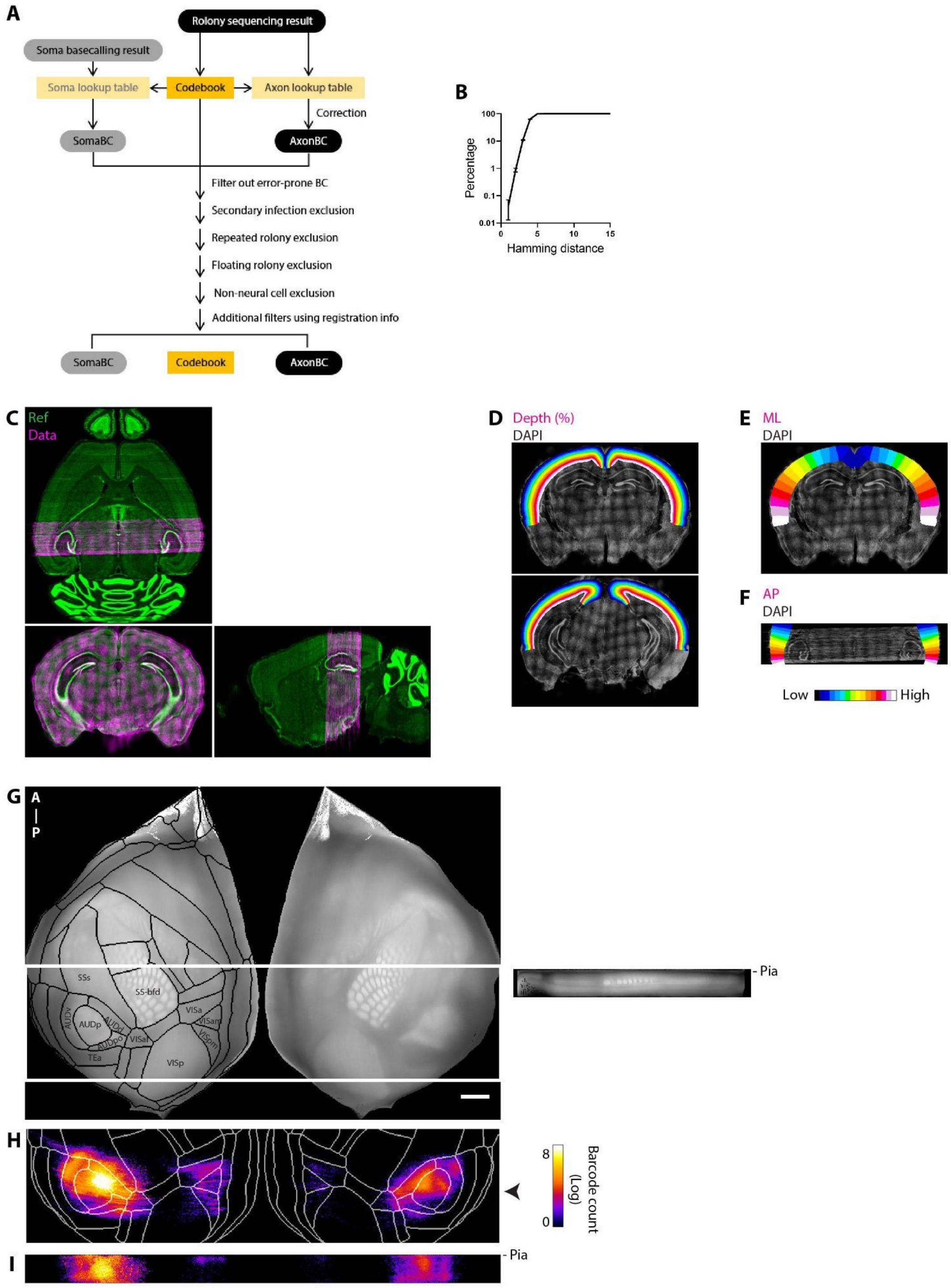

SupFig. 4 Barcode analysis and data registration. (**A**) The workflow of constructing a codebook and lookup tables for identifying somatic and axonal barcodes. (**B**) The possibility of barcodes having counterparts at various hamming distances. 10000 15-nt random generated barcode, mean ± SD from 100 iterations. (**C**) Representative plates showing the registration of the data to the Allen Brain Atlas common coordinate framework (CCFv3). Magenta, DAPI staining from registered data; green, Nissl staining of the reference atlas. (**D-F**) Representative images of cortical depth/ML/AP-value of the registered brain. Colormaps encode continuous gradients for depth/ML/AP-axis. E-F, coronal view; G, horizontal view. Gray, DAPI staining from the data. (**G**) Cortical flatmap

bioRxiv preprint doi: https://doi.org/10.1101/2023.02.18.528865; this version posted February 18, 2023. The copyright holder for this preprint (which was not certified by peer review) is the author/funder, who has granted bioRxiv a license to display the preprint in perpetuity. It is made available under aCC-BY 4.0 International license.

generated from CCFv3 average template. Grayscale: maximum intensity of the average template on ML-AP plate. White box: region of the axonal BARseq dataset. Area boundaries and annotation are shown in the left hemisphere. Area boundaries were drawn according to the most frequent area per pixel across cortical depth. Scale bar: 1 mm. Right panel, representative ML-depth view 125 μm slide of the right hemisphere. Top, cortical surface; bottom, the inner surface of the cortex. Depth percentage was converted to μm by assuming 1000 μm cortical thickness. (**H-I**) Distribution of barcodes on the flatmap. (H) ML-AP view. White, area boundaries; arrow, AP location of I. (I) Representative ML-depth view of barcode locations in cortex. Image included signals from 250 μm volume along the AP-axis.

bioRxiv preprint doi: https://doi.org/10.1101/2023.02.18.528865; this version posted February 18, 2023. The copyright holder for this preprint (which was not certified by peer review) is the author/funder, who has granted bioRxiv a license to display the preprint in perpetuity. It is made available under aCC-BY 4.0 International license.

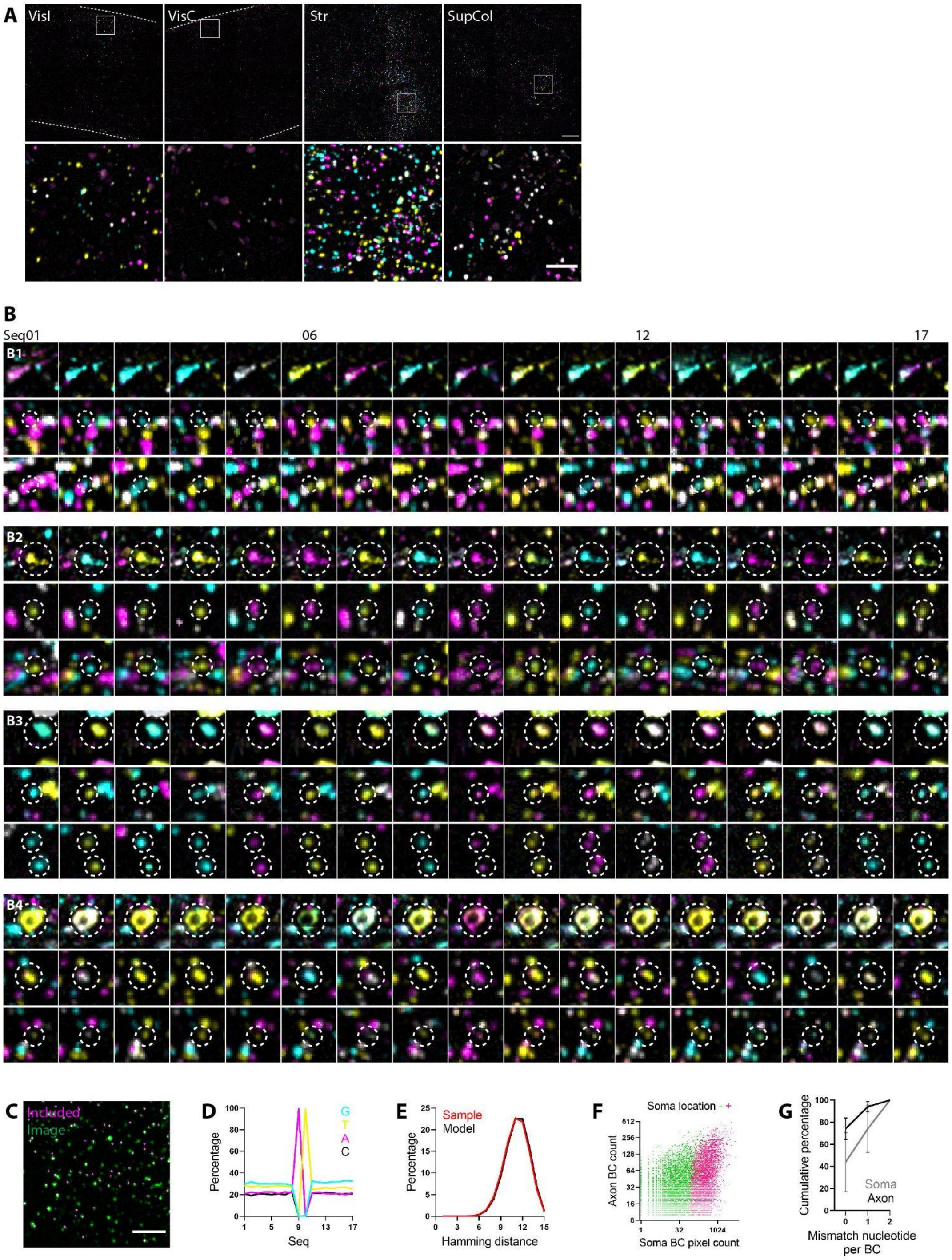

SupFig. 5 Example images and statistics of the dataset, related to Fig 1. (**A**) Representative images of axonal rolonies in VisI, VisC, Str and SupCol. Images are from Seq 01. Dotted line, cortical boundaries. Scale bar: top, 100 µm; bottom, 25 µm. Vis was identified according to CCFv3, it included posterior parietal cortex or parietal associative area (Lyamzin and Benucci 2019). (**B**) Additional examples of somatic and axonal rolonies from the same barcode are shown to illustrate the variability of sequencing signals in this dataset. Four examples are presented, with the top row showing images from somata and the middle and bottom rows showing images from axonal rolonies. The examples display differences in the size, shape, and intensity of axonal and somatic rolonies.

bioRxiv preprint doi: https://doi.org/10.1101/2023.02.18.528865; this version posted February 18, 2023. The copyright holder for this preprint (which was not certified by peer review) is the author/funder, who has granted bioRxiv a license to display the preprint in perpetuity. It is made available under aCC-BY 4.0 International license.

Some somata exhibit phasing (B3-4; phasing correction is described in SupFig. 3D), while others have clean signals (B2). In B2 and B3, duplications of the same rolonies were found in some sequencing cycles due to imperfect stitching, which was corrected in a later processing step (see Methods). (**C**) Example of the proportion of barcoded rolonies included in this dataset. Rolonies included in this dataset are indicated in magenta. Scale bar, 25 µm. (**D**) Nucleotide proportion of barcodes across sequencing cycles. The 9-10th nucleotides were fixed for this barcode library, 99.1% of the barcodes contained both fixed nucleotides. (**E**) The distribution of the all-to-all Hamming distance of the sample was similar to that of the model. Sample, 15-nt barcode without the two fixed nucleotides. Model, 10000 random generated 15-nt barcodes, mean ± SD of 100 iterations. (**F**) The relationship between the counts of somatic and axonal barcodes in individual barcoded cells. Cells were divided into two groups based on whether or not their soma location was identified. Cells that were somatic location-negative may still have barcodes detected in AudI, but their somatic locations were excluded from the analysis due to potential errors (see Methods). (**G**) Cumulative percentage of mismatch nucleotides per barcode in axon and soma. Barcodes with soma location were used to compute the soma barcode percentage; mean ± SD. Because the current codebook was constructed using axonal barcodes, it resulted in higher accuracy in identifying barcodes in axon compared to soma.

bioRxiv preprint doi: https://doi.org/10.1101/2023.02.18.528865; this version posted February 18, 2023. The copyright holder for this preprint (which was not certified by peer review) is the author/funder, who has granted bioRxiv a license to display the preprint in perpetuity. It is made available under aCC-BY 4.0 International license.

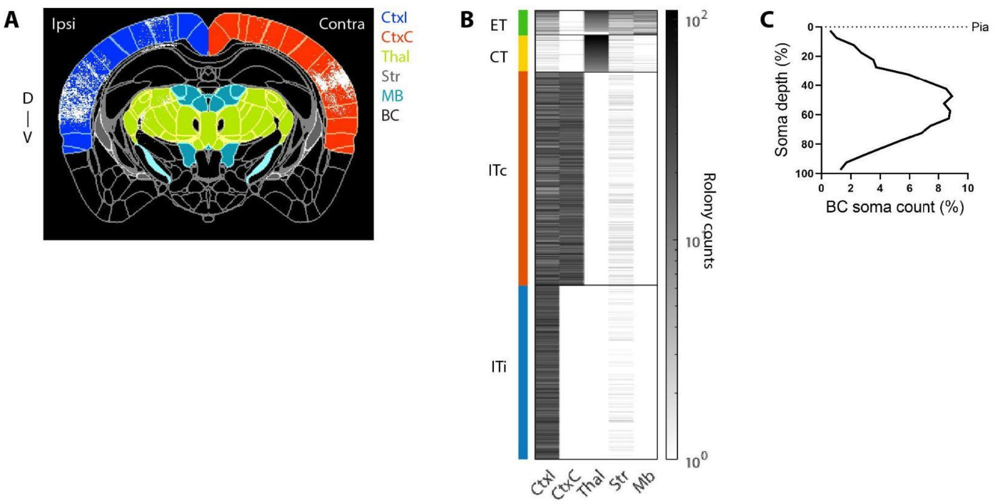

SupFig. 6 Projection targets of major cell types, related to Fig 3. (**A**) Representative coronal view of rolonies and five targeted brain regions. Rolonies located within the fiber bundles were not included in the five regions, 468026 of 492950 rolony were included. Target regions are color-coded; gray line: anatomical boundaries. CtxI, ipsilateral cortex; CtxC, contralateral cortex; Thal, thalamus; Str, striatum; MB, midbrain. (**B**) Heatmap of rolony counts in different targets from four cell types, ET, CT, ITc and ITi. (**C**) Distribution of barcoded somata along the cortical depth. Total 3698 barcoded somata. Bin size: 5% cortical depth.

bioRxiv preprint doi: https://doi.org/10.1101/2023.02.18.528865; this version posted February 18, 2023. The copyright holder for this preprint (which was not certified by peer review) is the author/funder, who has granted bioRxiv a license to display the preprint in perpetuity. It is made available under aCC-BY 4.0 International license.

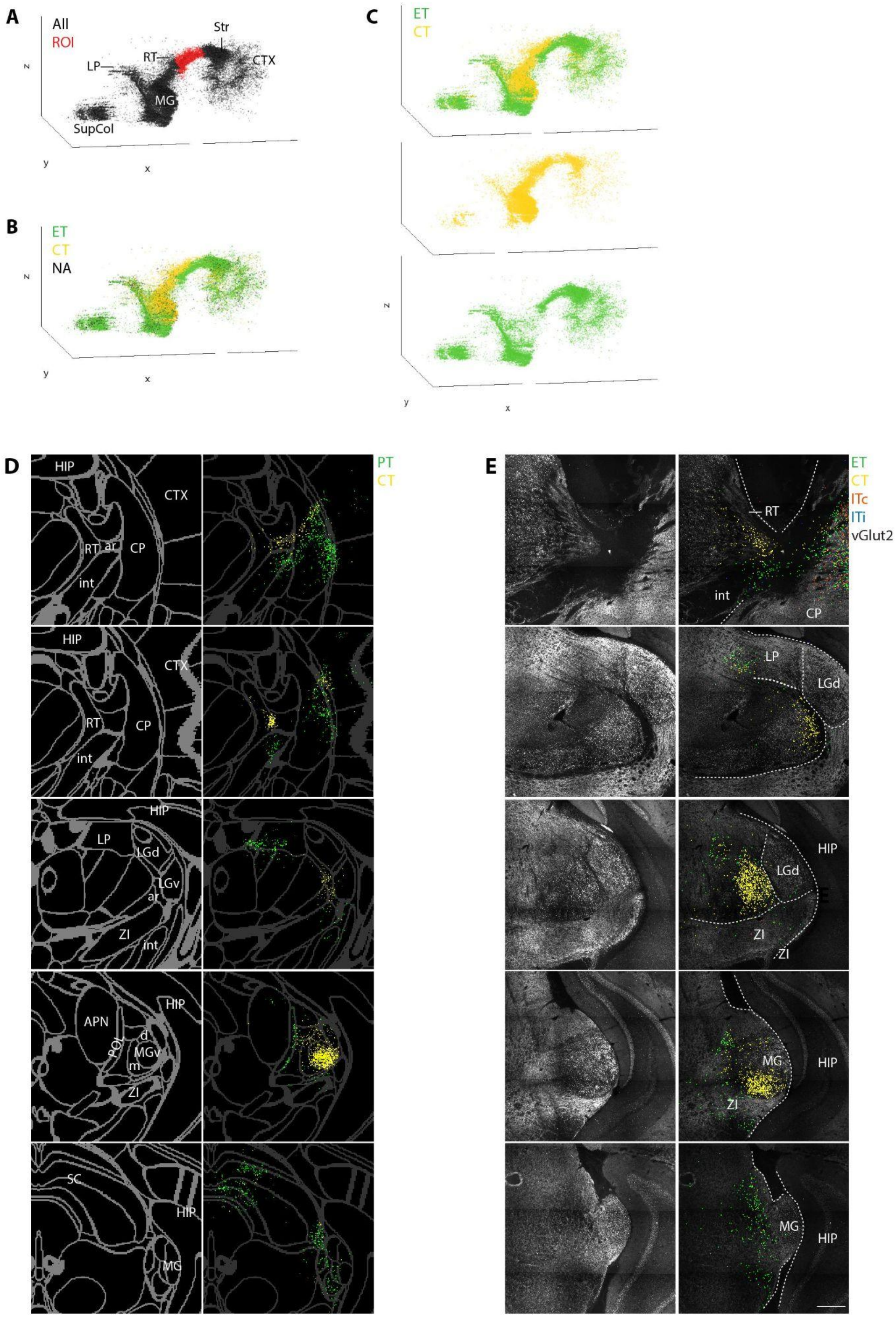

SupFig. 7 Division of CT and ET cells using projection targets and axonal trajectory, related to Fig 3. (**A-C**) The region of interest (ROI) and results from CT/ET grouping of Thal+ cells. (**A**) Side view of rolonies from Thal+ cells (black). Red, rolonies within the ROI (around RT and striatal-thalamic fiber tract), used for grouping CT and ET for Thal+ cells. (**B**) Initial grouping result using rolony within the ROI. Group identities were assigned to barcoded neurons according to their rolony locations within the ROI; individual rolonies were color-coded based on their cell identity. Black dots, rolonies from cells without rolony in ROI. (**C**) Final grouping result. Cells without rolony in the ROI were assigned to the group as their closest neighbors (see Methods). All Thal+ cells were assigned to either ET or CT. (**D**) Localization of registered CT and ET rolonies in striatum, thalamus and superior colliculus. Left column,

bioRxiv preprint doi: https://doi.org/10.1101/2023.02.18.528865; this version posted February 18, 2023. The copyright holder for this preprint (which was not certified by peer review) is the author/funder, who has granted bioRxiv a license to display the preprint in perpetuity. It is made available under aCC-BY 4.0 International license.

region boundaries with labels; right column, rolonies in the same region with tune-down boundaries for visualization. Each row is one coronal section, arranged from anterior (top) to posterior (bottom), from 6925, 7050, 7500, 8150 and 8775 µm in CCFv3, 25 µm/section. (**E**) Localization of CT and ET rolony with vGlut2 as an internal marker for thalamic structures. Left column, vGlut2 staining; right column, rolonies in the same region with tune-down vGlut2 signal for visualization. MG contains large but lower density of vGlut2 puncta (Hackett et al. 2016). To preserve the original resolution of vGlut2 staining, images and rolony locations were not registered. Each row is one coronal section, arranged from anterior (top) to posterior (bottom), from 220, 720, 1120, 1560 and 2020 µm in this dataset, 20 µm/section. Scale bar: 250 µm. CTX, cortex; CP, caudoputamen; ar, auditory radiation; RT, reticular nucleus; int, internal capsule/cerebral peduncle; HIP, hippocampus; LP, lateral posterior nucleus; LG(d/v), lateral geniculate complex (dorsal/ventral part); APN, anterior pretectal nucleus; POL, posterior limiting nucleus; MG(v/d/m), medial geniculate complex (ventral/dorsal/medial part); ZI, zona incerta; SC, superior colliculus.

bioRxiv preprint doi: https://doi.org/10.1101/2023.02.18.528865; this version posted February 18, 2023. The copyright holder for this preprint (which was not certified by peer review) is the author/funder, who has granted bioRxiv a license to display the preprint in perpetuity. It is made available under aCC-BY 4.0 International license.

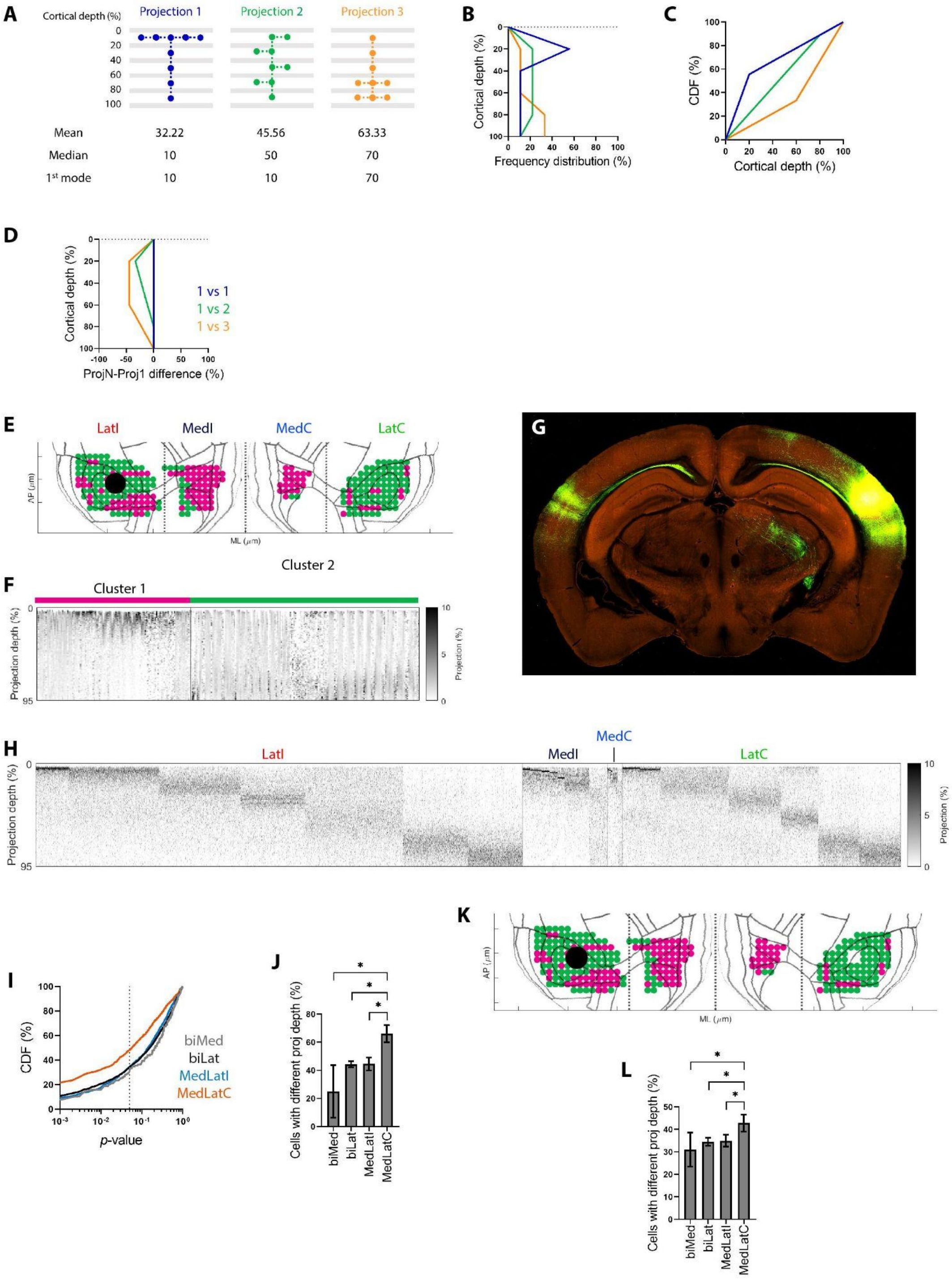

SupFig. 8 Description and comparison of laminar patterns, related to Fig 4. (**A-D**) Example for representing and comparing cortical laminar patterns. (**A**) Diagram of three projections with distinct laminar patterns across the cortical depth. Dots represent rolonies and gray horizontal lines indicate cortical depth with 20% intervals. Projection 1 primarily projects to the superficial layer, projection 2 spreads across layers, and projection 3 primarily projects to the lower layers. The mean, median, and mode depths for each projection are listed below. Using a single measurement to describe laminar patterns can create bias in some cases. For example, median may be a better measurement than mean for a Poisson distribution, but it may not be as accurate for a multinomial distribution. Here, we described projection laminar patterns using frequency distribution (**B**) and cumulative distribution (**C**). (**D**)

bioRxiv preprint doi: https://doi.org/10.1101/2023.02.18.528865; this version posted February 18, 2023. The copyright holder for this preprint (which was not certified by peer review) is the author/funder, who has granted bioRxiv a license to display the preprint in perpetuity. It is made available under aCC-BY 4.0 International license.

Pairwise comparison of cumulative distribution of rolony depth. Each line represents the distribution difference (x-axis) at the cortical depth (y-axis). For projection 1 vs. projection 1 comparison, all the data points are 0 across depth due to both projections being completely the same. (**E-F**) Different laminar patterns of bulk projections in medial and lateral cortical targets. Data were computed and plotted using cortical flatmap. Cortical rolonies were binned into 250 × 250 μm bins on ML-AP plate; frequency distributions of rolony depth were calculated for each bin (1% depth per bin). Bins were split into two clusters using k-means and color-coded as magenta and green. Minimum 50 rolonies per bin, total 301 bins. (**E**) Spatial location of these bins on flatmap. Each bin is indicated by a dot and color-coded by cluster number. (**F**) Frequency distribution of rolony depth for all bins in each cluster, one bin per column. (**G**) Representative AUD bulk tracing result from C57BL/6J (experiment 158314278) from Allen Connectivity Atlas (Oh et al. 2014). Additional AUD tracing results from C57BL/6J (115958825, 146858006) and Emx1-IRES-Cre (576332845, 554421791, 562671482). (**H**) Different laminar patterns of single-cell projections in medial and lateral cortical targets. One cell per column; cell counts for each area: LatI, 6878; MedI, 1196; MedC, 212; LatC, 3931. F-G, bin size, 1% depth. (**I**) The possibility of similar laminar projection patterns in two different areas originating from a single cell. The possibilities were represented by *p*-values. The distributions were plotted as cumulative distribution functions. Dotted line: 0.05. (**J**) Control analysis for Fig. 4E, with minimum 15 rolonies per cortical target. Cell counts: biMed, 16; biLat, 1834; MedLatI, 484; MedLatC, 245. (**K-L**) The laminar pattern differences in the dataset were shown, with rolonies in the mirror somatic region of LatC excluded (LatC local-exclusion control). (**K**) Spatial locations of the two types of cortical bins as SupFig. 8E, the corresponding soma region in LatC is empty due to data exclusion. Total 297 bins. (**L**) The proportion of cells with different laminar patterns as Fig. 4E. *, significant difference with no overlap CI.

bioRxiv preprint doi: https://doi.org/10.1101/2023.02.18.528865; this version posted February 18, 2023. The copyright holder for this preprint (which was not certified by peer review) is the author/funder, who has granted bioRxiv a license to display the preprint in perpetuity. It is made available under aCC-BY 4.0 International license.

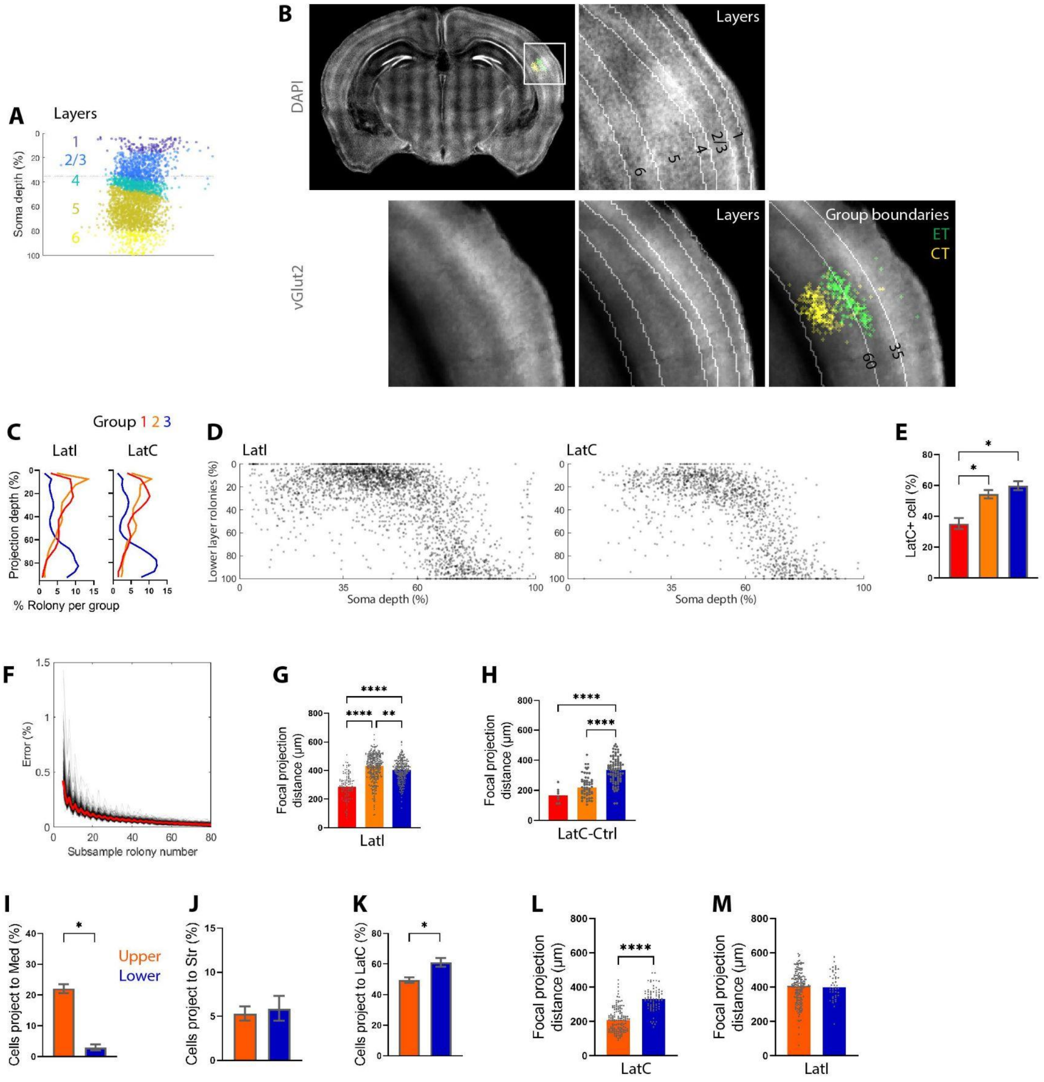

SupFig. 9 Projection differences across IT cell groups, related to Fig 5. (**A**) Layer annotations of Lat-projecting IT cells in Fig. 5A. Flatmap view. Dotted line: 35 and 60% depth. Layer annotations are from CCFv3. (**B**) Coronal view of the laminar boundaries of the three groups with annotations and internal markers. Zoom-in view: injection site. Top row: DAPI; bottom row: vGlut2. The 35% boundary approximately corresponds to the upper boundary of layer 4, which has relatively strong vGlut2 and DAPI signals (Nahmani and Erisir 2005; Coleman et al. 2010; Sato et al. 2022). The 60% boundary corresponds to the peak of the ET neuron distribution (green, also see Fig. 3C), and the weak vGlut2 band in upper layer 5 (Hirai et al. 2012; Oswald et al. 2013). The Images were generated from 312.5 µm volume of the registered data. Soma positions are sum projections and markers are median projections of the volume. (**C**) At bulk level, group 3 Lat-projecting cells mainly projected to lower layers while group 1/2 cells projected to all layers with preference to upper-middle layers. Lines represent the frequency distribution of rolony depth per group. Bin size, 5% depth. (**D**) Gradual increase of lower layer projection at single-cell level in Lat. One cell is indicated by a dot. (**E**) Group 1 has a lower percentage of cells projected to contralateral Lat cortex. Bar graph, median and confidence intervals. *, significant difference with no overlap CI. (**F**) Effect of rolony number on computing focal projection distance. LatC-projecting cells with minimum 80 rolonies were used for testing, and 5-80 rolonies were randomly sampled per cell. The error caused by downsampling was calculated using the distance difference between downsampling and ground truth (see Methods). Ground truth distance was represented by distance computed with all rolonies. Black line, median error from each barcoded cell, total 314 cells; solid red line, median of black lines. The median error < 5% when sample rolony number ≥ 55, therefore, the minimum rolony

bioRxiv preprint doi: https://doi.org/10.1101/2023.02.18.528865; this version posted February 18, 2023. The copyright holder for this preprint (which was not certified by peer review) is the author/funder, who has granted bioRxiv a license to display the preprint in perpetuity. It is made available under aCC-BY 4.0 International license.

number for this test was set to 55. (**G-H**) Projections from group 1 Lat-projecting cells were more focal compared to group 3 in LatI (G) and in LatC local-exclusion control (H). Cell counts: LatI group1 117, group2 286, group3 256; LatC-Ctrl group1 8, group2 56, group3 93. Kruskal-Wallis test, Dunn's test for multiple comparisons. (I-M) Morphological difference between lower and upper layer-projecting cells without soma location information. Lat-projecting cells were split into upper/lower layer-projecting cells using median depth in LatI/C: upper, ≤ 60%, lower, > 60%. In 1958 biLat cells, 209 (10.7%) were assigned to different groups in two Lat regions and they were excluded. Cell counts: upper, 2993; lower, 1025. Consistently, upper layer-projecting cells had a higher percentage of cells projected to Med (I), a lower percentage of cells projected to LatC (K), and more focal projections in LatC (L). Due to upper layer-projecting cells consisting of group 1 and 2 neurons, no difference was found in (J) and (M). I and K, *, significant difference with no overlap CI. L-M, Mann-Whitney test.

bioRxiv preprint doi: https://doi.org/10.1101/2023.02.18.528865; this version posted February 18, 2023. The copyright holder for this preprint (which was not certified by peer review) is the author/funder, who has granted bioRxiv a license to display the preprint in perpetuity. It is made available under aCC-BY 4.0 International license.

Supplementary tables

SupTable 1, List of brain sections and imaged areas

SupTable 2, List of reagents and optics

Supplementary video

SupVideo, Single-cell reconstruction from 100 barcoded neurons
